# Supplementary material for: Complexome profiling of the Chlamydomonas psb28 mutant reveals TEF5 as an early PSII assembly factor
Source: Plant Cell. 2025 Mar 17;37(6):koaf055. doi: 10.1093/plcell/koaf055 (PMC12164590; doi:10.1093/plcell/koaf055)
Supplement: koaf055_Supplementary_Data [file koaf055_supplementary_data.zip › psb28_interactive.zip!/plots/intensity/qualityPlots/peptides/MDN5_Cre09.g410700.t1.2.html]

### Cre09.g410700.t1.2

Trivial name: MDN5  
SeqCoverage [median]: 13.30  
Mass: 44.85 kDa  
  
 

| Sequence | MissedCleavage | Mass [Da] | wtIntensity | muIntensity | Score |
| --- | --- | --- | --- | --- | --- |
| ALNESASR | 0 | 846.42 | 0.00 | 1021.30 | 81.30 |
| FYTSVSR | 0 | 858.42 | 400.24 | 0.00 | 87.28 |
| IGGLPAPDVIR | 0 | 1106.64 | 0.00 | 2314.87 | 55.55 |
| LSYDTQNEDASLTR | 0 | 1611.74 | 729.23 | 1855.82 | 169.89 |
| SSAASTAVSVADAIR | 0 | 1404.72 | 1762.23 | 5625.86 | 153.34 |
|  |  |  | 2891.7 | 10817.9 |  |
